# Tongguan capsule for treating myocardial ischemia-reperfusion injury: integrating network pharmacology and mechanism study

**DOI:** 10.1080/13880209.2023.2175877

**Published:** 2023-02-15

**Authors:** Jiantao Liu, Chunping Liu, Huiqi Chen, Huan Cen, Hailong Yang, Peijian Liu, Fang Liu, Liuling Ma, Quanfu Chen, Lei Wang

**Affiliations:** a State Key Laboratory of Dampness Syndrome of Chinese Medicine, The Second Affiliated Hospital of Guangzhou University of Chinese Medicine, Guangzhou, China; b Shunde Hospital of Guangzhou University of Chinese Medicine, Foshan, China; c Guangdong-Hong Kong-Macau Joint Lab on Chinese Medicine and Immune Disease Research, Guangzhou, China; d State Key Laboratory of Quality Research in Chinese Medicine, Institute of Chinese Medical Sciences, University of Macau, Macau, China; e School of Pharmaceutical Sciences, Guangzhou University of Chinese Medicine, Guangzhou, China; f Department of Cardiovascular Medicine, The Second Affiliated Hospital of Guangzhou University of Chinese Medicine, Guangzhou, China

**Keywords:** Hypoxia/reoxygenation, apoptosis, mTOR pathways, autophagy

## Abstract

**Context:**

Although Tongguan capsule (TGC) is used in the treatment of coronary atherosclerotic disease, the exact mechanism remains unclear.

**Objective:**

Network pharmacology and experimental validation were applied to examine the mechanism of TGC for treating myocardial ischemia-reperfusion injury (MIRI).

**Materials and methods:**

The components and candidate targets were searched based on various databases such as TCMSP, TCMID, BATMAN-TCM. The binding ability was determined by molecular docking. The ischemia-reperfusion (I/R) model was constructed by ligating the left anterior descending (LAD) coronary artery. APOE^-/-^ mice were divided into three groups (*n* = 6): Sham group, I/R group, and TGC group (1 g/kg/d). To further verification, HCAEC cells were subjected to hypoxia-reoxygenation (H/R) to establish *in vitro* model.

**Results:**

The compounds, such as quercetin, luteolin, tanshinone IIA, kaempferol and bifendate, were obtained after screening. The affinity values of the components with GSK-3β, mTOR, Beclin-1, and LC3 were all <-5 kcal/mol. *In vivo*, TGC improved LVEF and FS, reducing infarct size. *In vitro*, Hoechst 33258 staining result showed TGC inhibited apoptosis. Compare with the H/R model, TGC treatment increased the levels of GSK-3β, LC3, and Beclin1, while decreasing the expression of mTOR and p62 (*p* < 0.05).

**Discussion and Conclusion:**

The findings revealed that TGC exerted a cardioprotective effect by up regulating autophagy-related proteins through the mTOR pathway, which may be a therapeutic option for MIRI. However, there are still some limitations in this research. It is necessary to search more databases to obtain information and further demonstrated through randomized controlled trials for generalization.

## Introduction

Reperfusion therapy is a milestone achievement in the management of acute myocardial infarction (AMI) (Lee and Hanif 2018). Early reperfusion therapy can restore blood flow through the occluded coronary artery immediately upon diagnosis, thus protecting heart function and improving the prognosis of patients with AMI (Vogel et al. 2019). However, even if the arterial stenosis or occlusion is relieved, some patients experience slow coronary blood flow, suggesting cardiac microvascular disorder (Kumar et al. 2019; Wang et al. 2020a, 2020b). In addition, ischemia-reperfusion (I/R) causes functional damage of endothelial cells, which is the crucial mechanism of microvascular diseases (Zhou and Toan 2020; Wang et al. 2020a). Therefore, endothelial homeostasis is critical for alleviating myocardial ischemia-reperfusion injury (MIRI) (Davidson et al. 2019).

Previous studies have shown that traditional Chinese medicine (TCM) may have its own unique role in the treatment of MIRI (Wang et al. 2020c; Xie et al. 2021). Tongguan capsule (TGC), a proprietary Chinese medicine, composed of *Astragalus membranaceus* Moench (Fabaceae), *Salvia miltiorrhiza* Bunge (Lamiaceae), Borneol, and Leech, play the role on promoting blood circulation, invigorating *qi*, replenishing energy and improving metabolism in TCM (Shuai et al. 2018; Ma et al. 2019a). Based on accurate fingerprint analysis with high-performance liquid chromatography, TGC contains substantial active compounds, such as *tanshinone* IIA and *salvianolic* acid B (Mao et al. 2020). TGC improves cardiac function in patients with coronary atherosclerotic disease, inhibiting postoperative ventricular remodeling and vascular restenosis (Shuai et al. 2018; Ma et al. 2019; Zhou et al. 2021).

**Figure 1.**
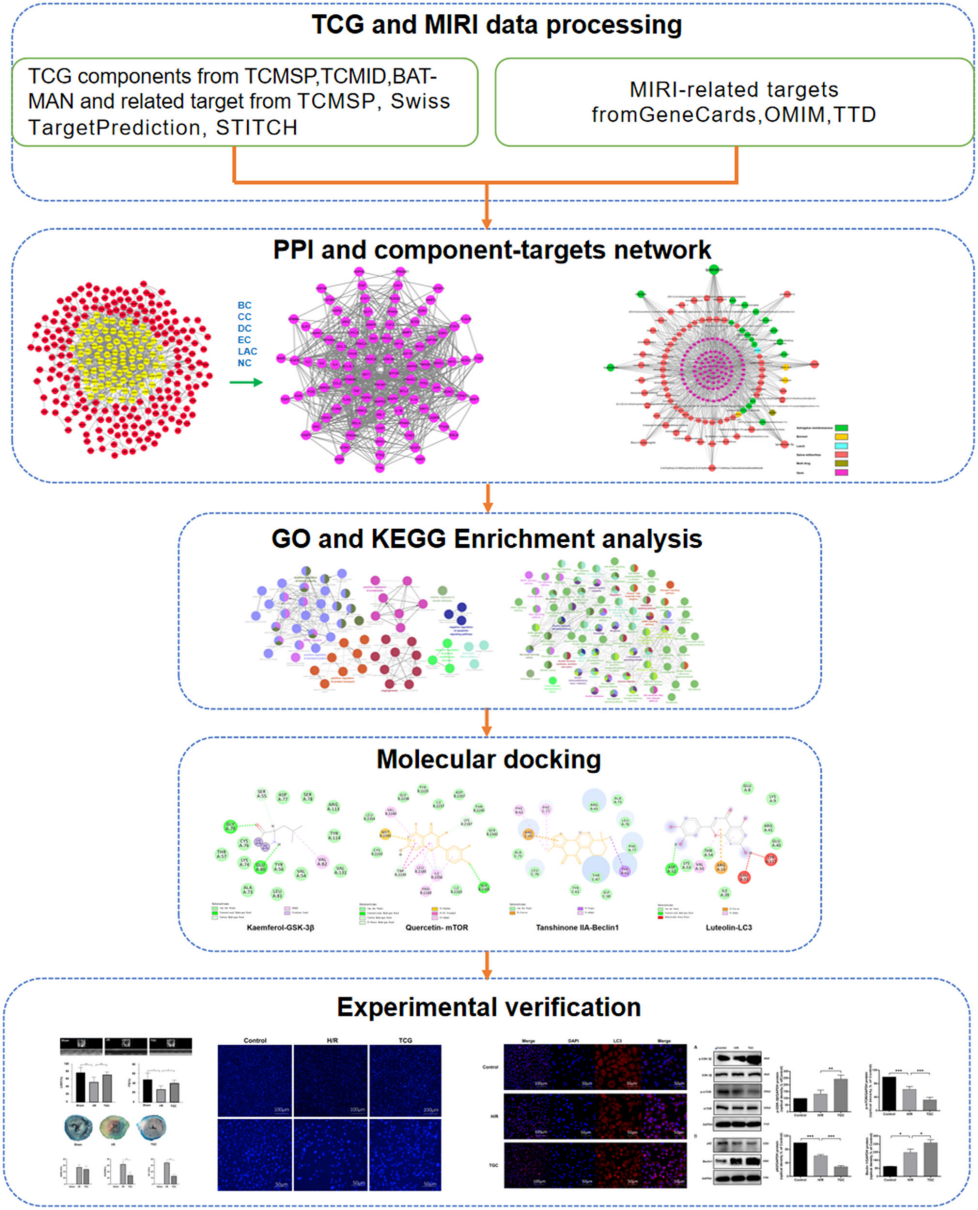
Workflow for TGC in the treatment of MIRI.

However, the molecular mechanism of TGC is unclear, which needs to be preliminarily explored through network pharmacology. Network pharmacology, based on bioinformation data analysis, is devoted to discover the complex mechanisms of drugs by searching for components and targets (Kibble et al. 2015; Muhammad et al. 2018; Zhang et al. 2019a). Molecular docking is applied to the study of interactions between ligands and receptors by simulating the binding (Pinzi and Rastelli 2019). In this study, compounds and targets were screened through network pharmacological analysis, verified by molecular docking and experiment, to elucidate the mechanism of TGC for treating MIRI, providing a reference for clinical medication. The workflow of this study was shown in Figure 1.

## Materials and methods

### Active ingredient screening

The TGC active ingredients, with drug-likeness (DL) ≥0.18 and oral bioavailability (OB) ≥30%, were screened from TCMSP (https://tcmspw.com/tcmsp.php), TCMID (http://119.3.41.228:8000/tcmid/) and BATMAN-TCM (http://bionet.ncpsb.org.cn/batman-tcm/). The targets of the compounds were obtained through TCMSP, Swiss TargetPrediction (http://swisstargetprediction.ch/), and STITCH (http://stitch.embl.de/). Simultaneously, the UniProt database (https://www.uniprot.org/) was applied for gene name standardization.

### Identification of candidate targets

The targets related to ‘myocardial ischemia-reperfusion injury’ were retrieved through the GeneCards (https://www.genecards.org), Online Mendelian Inheritance in Man (OMIM, https://omim.org), and TTD (http://db.idrblab.net/ttd/). The overlapping genes with MIRI and active components were identified as candidate targets.

### PPI network construction

The candidate targets were imported into String (https://string-db.org/), and the condition was set as *Homo sapiens* with a confidence of 0.95 to obtain the relevant information on protein interactions. PPI network was constructed through Cytoscape (V3.7.1) and CytoNCA plug-in was devoted to calculate the topology index, including betweenness centrality (BC), closeness centrality (CC), degree centrality (DC), eigenvector centrality (EC), local average connectivity-based method (LAC), and network centrality (NC). The genes greater than the median of each topological index were regarded as core targets.

### Component-target network

The network between active components and core targets was visualized by Cytoscape, from which the important compounds were obtained according to the degree value.

### Enrichment analysis

With *p* value < 0.05 and κ set to 0.4, under the conditions of *Homo sapiens*, the ClueGO plug-in of Cytoscape was applied for Gene Ontology (GO) and Kyoto Encyclopedia of Genes and Genomes (KEGG) enrichment analysis of the core targets.

### Molecular docking

The 2D structures of important compounds were obtained from PubChem (https://pubchem.ncbi.nlm.nih.gov/), and 3D chemical structures were created by ChemBioDraw software. Protein structures were searched from PDB (https://www.rcsb.org/) according to UniProt ID. Water molecules and ligands of protein were removed by Discovery Studio. AutoDock Vina software was used for molecular docking with the binding site of the protein’s own ligand as the active pocket.

### Animal model of I/R

Male ApoE^-/-^ mice weighing 20 ± 2 g were obtained from Guangdong Medical Experimental Animal Center. The animals were housed in an SPF environment with a temperature of 22–25 °C, relative humidity of 60%, and 12 h light/dark cycle, with free access to adequate water and food. All procedures (including the mouse euthanasia) were performed in compliance with the regulations of the Second Affiliated Hospital of Guangzhou University of Chinese Medicine institutional animal care (Ethical number: 2016026) and conducted according to the AAALAC and IACUC guidelines (Ingham et al. 2000).

The operations were completed in the Laboratory Animal Center of the Second Affiliated Hospital of Guangzhou University of Traditional Chinese Medicine. Mice were randomly divided into 3 groups (*n* = 6): Sham group, I/R group, and TGC group (1 g/kg/d). During I/R surgery, the left anterior descending (LAD) coronary artery was ligated with 8–0 nylon suture. The ST segment elevation on the electrocardiogram and the color change of myocardial tissue indicated that myocardial ischemia was successfully established. Sutures were released after 45 min to induce 24 h of reperfusion. Mice in sham group underwent similar surgery without LAD coronary artery occlusion. All mice were given intragastric administration 5 days before surgery. The TGC group was administered TGC (Lot number: 161201, obtained from Guangdong Provincial Hospital of Chinese Medicine), while the sham group and the I/R group were administered the same volume of normal saline.

### Echocardiography

The mice were anesthetized with 2% isoflurane (VETEASY, China), and heart function was assessed by transthoracic echocardiography (Vevo 2100, Canada). After measurement, left ventricular ejection fraction (LVEF) and fractional shortening (FS) were recorded.

### Evans blue-TTC staining

After echocardiography was completed, the infarct size of the heart was determined by Evans blue-TTC staining. Briefly, the LAD coronary artery was sutured, and Evans blue solution (Meilunbio, China) was injected into heart vessels through the aorta. The heart was quickly removed and snap-frozen at −20 °C for 30 min, and then was cut into 1 mm thick slices and placed into TTC solution (Sigma-Aldrich, USA) at 37 °C for 15 min. The sections were fixed with a 4% paraformaldehyde solution. Image-Pro Plus software was devoted to assess infarct size (IA), area at risk (AAR), and total left ventricular area (LV), from which IA/AAR and IA/LV were calculated.

### Cell model of hypoxia/reoxygenation (H/R)

HCAECs from BeNa Culture Collection, China, were cultured in DMEM (Gibco, China) supplemented with 10% FBS (HyClone, USA) and 1% penicillin/streptomycin in a humidified atmosphere containing 5% CO_2_/95% air at 37 °C. Before the experiment, the cells were regularly subcultured until they reached 80% confluence.

Cells were then divided into a control group, H/R group, and TGC group. Cells in H/R group were cultured in glucose-free medium with hypoxia (94% N_2_, 5% CO_2_, 1% O_2_) for 3 h and then transferred to 10% fetal bovine serum (FBS, HyClone, USA) medium in a normoxic incubator for 1h. Cell in TGC group was treated with 100 μg/mL TGC solution for 7 h before oxygen-glucose deprivation (OGD). Cells in the control group were cultured under normal conditions.

### Hoechst 33258 staining and immunofluorescence

After the medium was discarded, the cells were washed with PBS (HyClone USA) 2 times and rapidly immobilized with 4% paraformaldehyde. The Hoechst 33258 solution (Beyotime, China) was then used to stain the cells for 15 min at room temperature. The images were observed under a fluorescence microscope (Nikon, China).

When performing immunofluorescence, the cell washing and immobilization steps were similar to those in Hoechst 33258 staining. Then, the cells were permeabilized in 0.5% Triton X-100 for 5 min and sealed in 5% BSA for 2 h. After being incubated with LC3 primary antibody (CST, USA) at 4 °C overnight, the cells were treated with fluorescently labeled secondary antibody (Invitrogen, USA) and DAPI (BOSTER, China) at room temperature. The images were observed under a fluorescence microscope, and the expression level was analyzed based on the fluorescence intensity.

### Western blot

After H/R, the cells were dissolved in RIPA buffer containing protease and phosphoprotein inhibitors. A BCA kit (Thermo Fisher Scientific, USA) was used to detect the protein content. The protein samples were then separated by sodium dodecyl sulfate-polyacrylamide gel electrophoresis and transferred to a polyvinylidene fluoride (PVDF) membrane. After blocking with 5% bovine serum albumin, the PVDF membrane was incubated with primary antibody at 4 °C overnight and with secondary antibody (CST, USA) at room temperature for 1 h. The primary antibodies were GAPDH (CST, USA), GSK-3beta (CST, USA), P-GSK-3β (CST, USA), mTOR (CST, USA), P-mTOR (CST, USA), Beclin1 (CST, USA), and p62 (CST, USA). The band development was performed with an ECL kit (Millipore, USA) and the results were analyzed by Image J software.

### Statistical analysis

All data were expressed as the mean ± SD and statistically analyzed by GraphPad Prism 8 software. Statistical significance analysis was performed using one-way ANOVA. The difference was considered statistically significant at *p* < 0.05.

## Results

### Identification of active compounds and candidate targets

**Figure 2.**
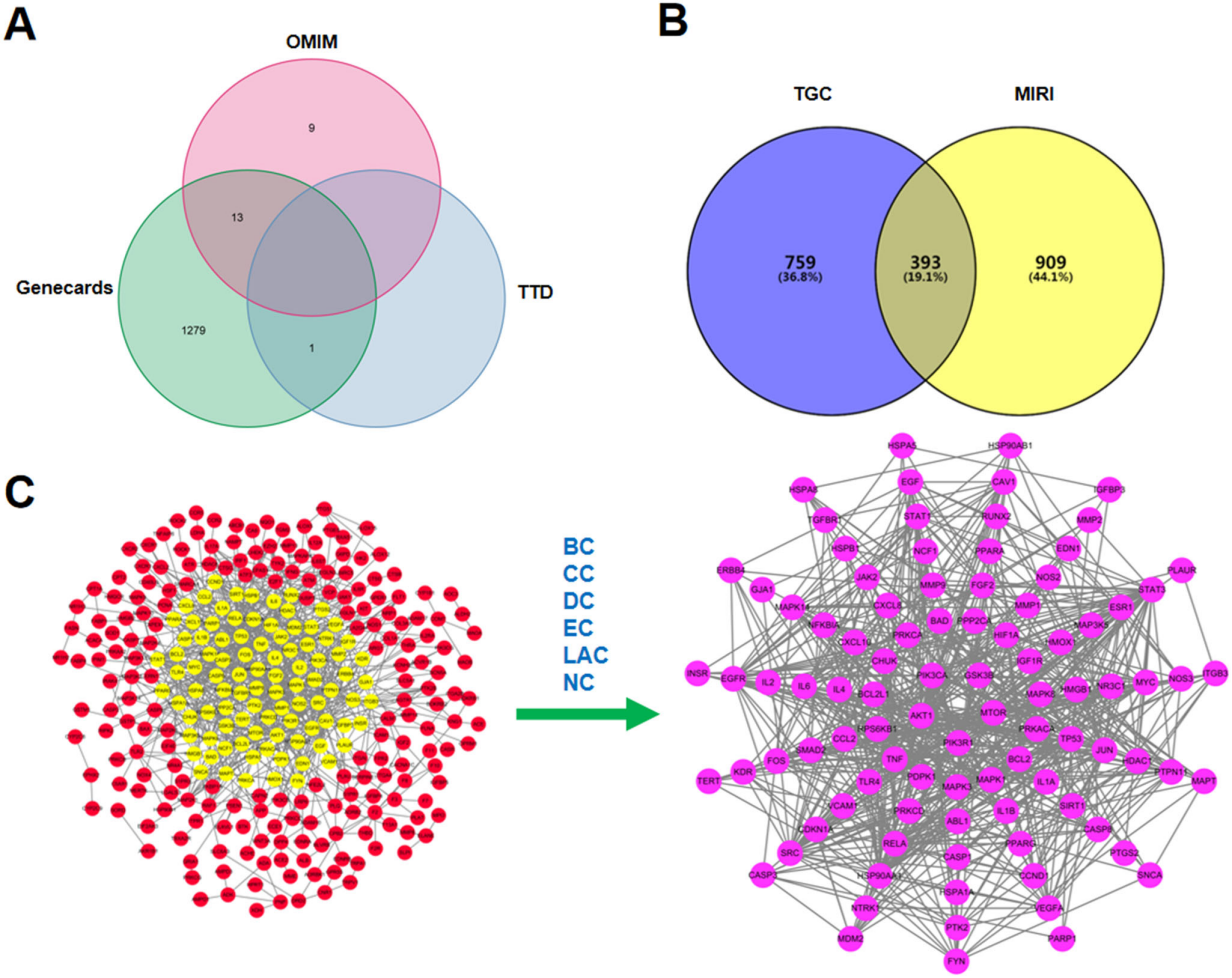
Acquisition of core targets. (A) MIRI-related targets identified based on GeneCards, OMIM, and TTD. (B) The overlapping genes both in TGC and MIRI are candidate targets. (C) 95 core targets were obtained according to BC, CC, DC, EC, LAC, and NC.

Based on TCMSP, TCMID, and BATMAN, 91 active compounds met the screening conditions. A total of 1143 targets of the active compounds were searched from TCMSP, Swiss TargetPrediction, and STITCH. 1302 MIRI-related targets were identified according to GeneCards, OMIM, and TTD. A total of 393 overlapping genes both in MIRI and TGC were recognized as candidate targets. By calculating the median of topological indexes, 95 core targets were obtained, including mTOR, GSK-3β, AKT1, PIK3CA, and PIK3R1 (Figure 2).

### Component-target network

**Figure 3.**
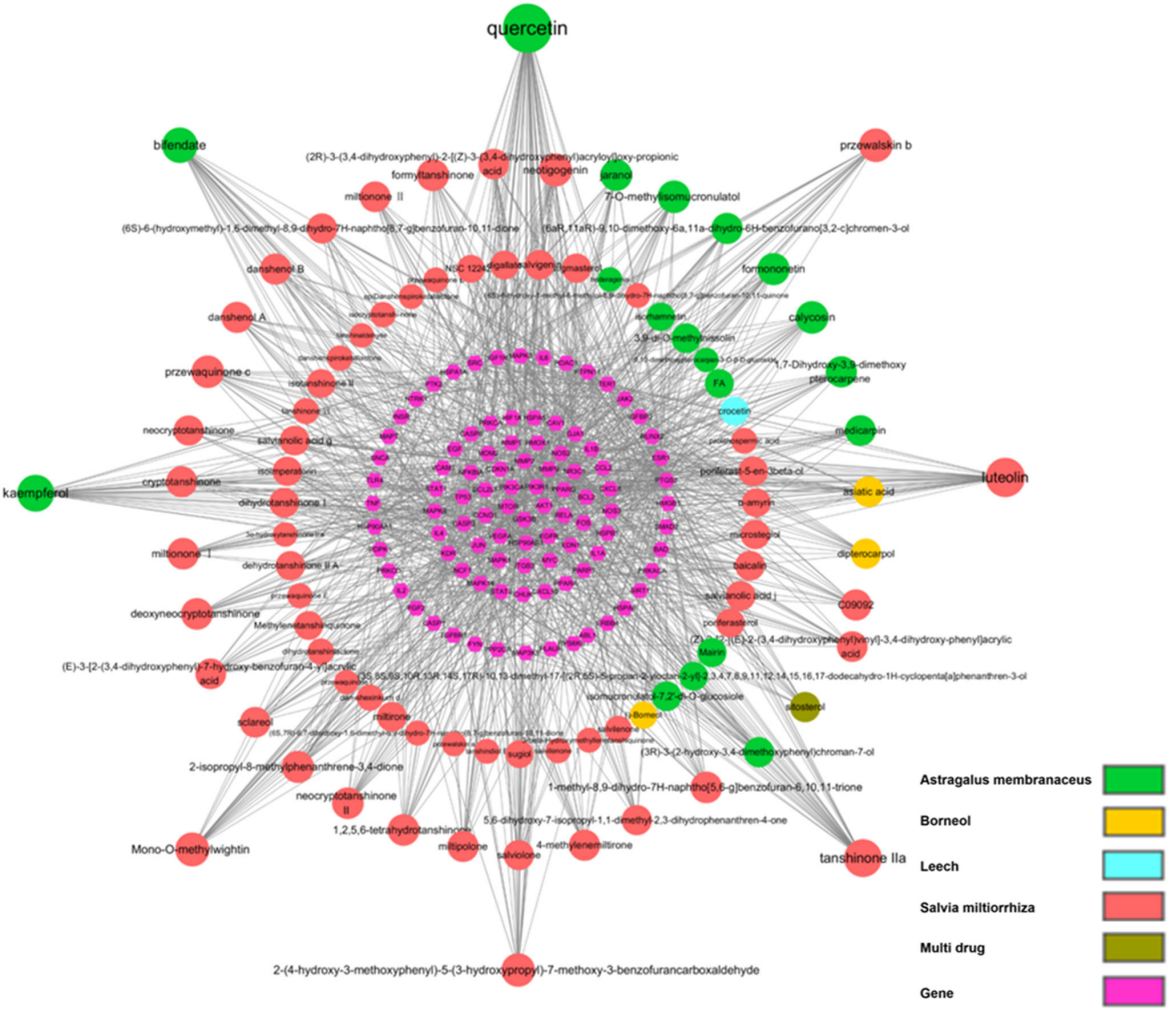
Component-target network. The pink hexagon nodes refer to the target, and the circular nodes of different colors represent the compound of various traditional Chinese medicines. The size of circular nodes reflects the degree of the component.

In the network (Figure 3), 90 components in TGC were correlated with the core targets. There are eight active components with degree >20, which are considered as important compounds, including *quercetin*, *luteolin*, *tanshinone* IIA, *kaempferol*, and *bifendate*.

### Enrichment analysis

**Figure 4.**
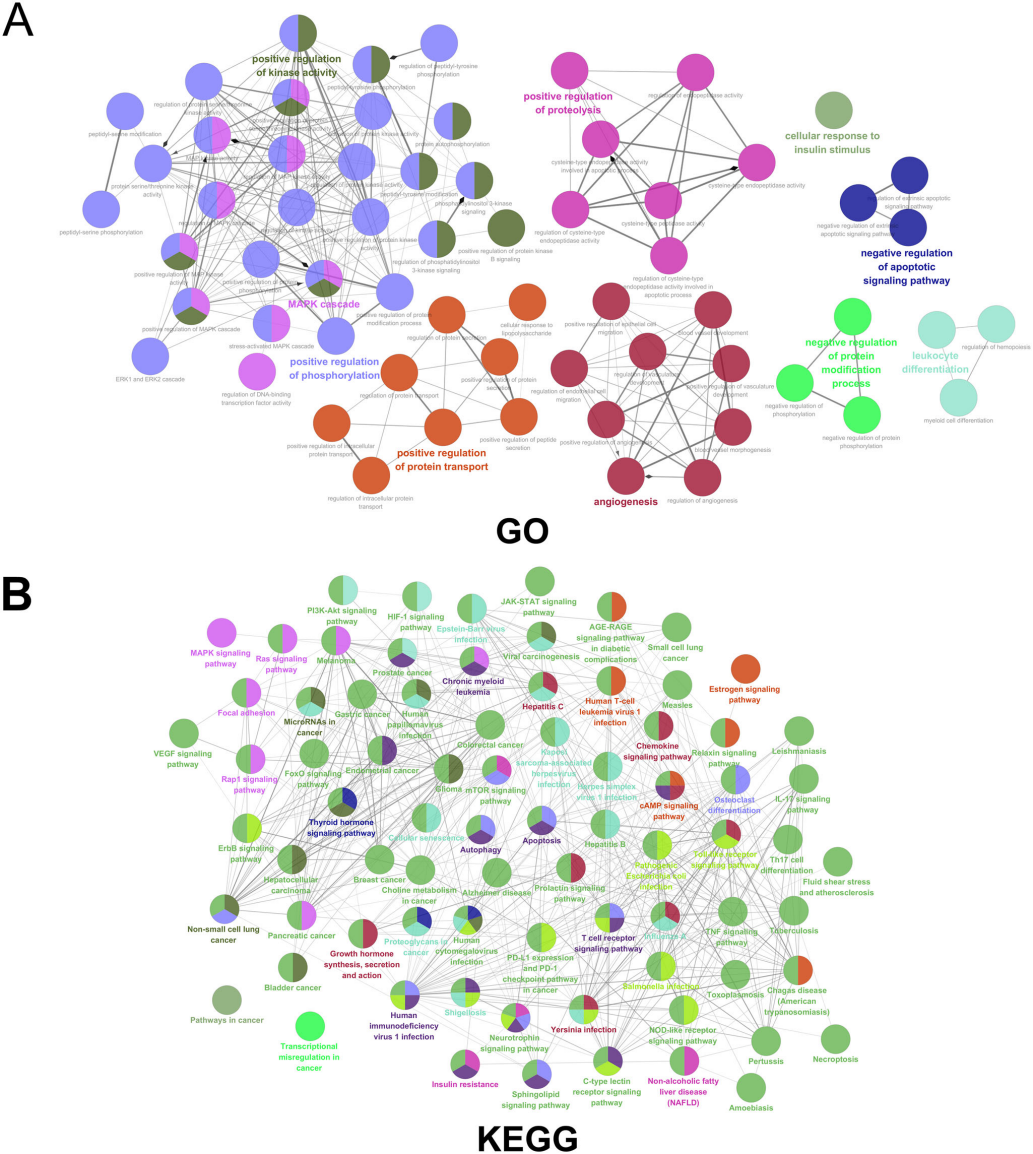
GO and KEGG enrichment analysis.

GO enrichment analysis (Figure 4(A)) indicated that the targets were mainly enriched in positive regulation of phosphorylation, positive regulation of kinase activity, angiogenesis, positive regulation of protein transport, and negative regulation of apoptotic signaling pathway. The KEGG results (Figure 4(B)) further suggested that TGC in the treatment of MIRI might be related to the PI3K − AKT signaling pathway and mTOR signaling pathway, which were crucial in autophagy and apoptosis.

### Molecular docking of compounds with proteins in the mTOR pathway

**Figure 5.**
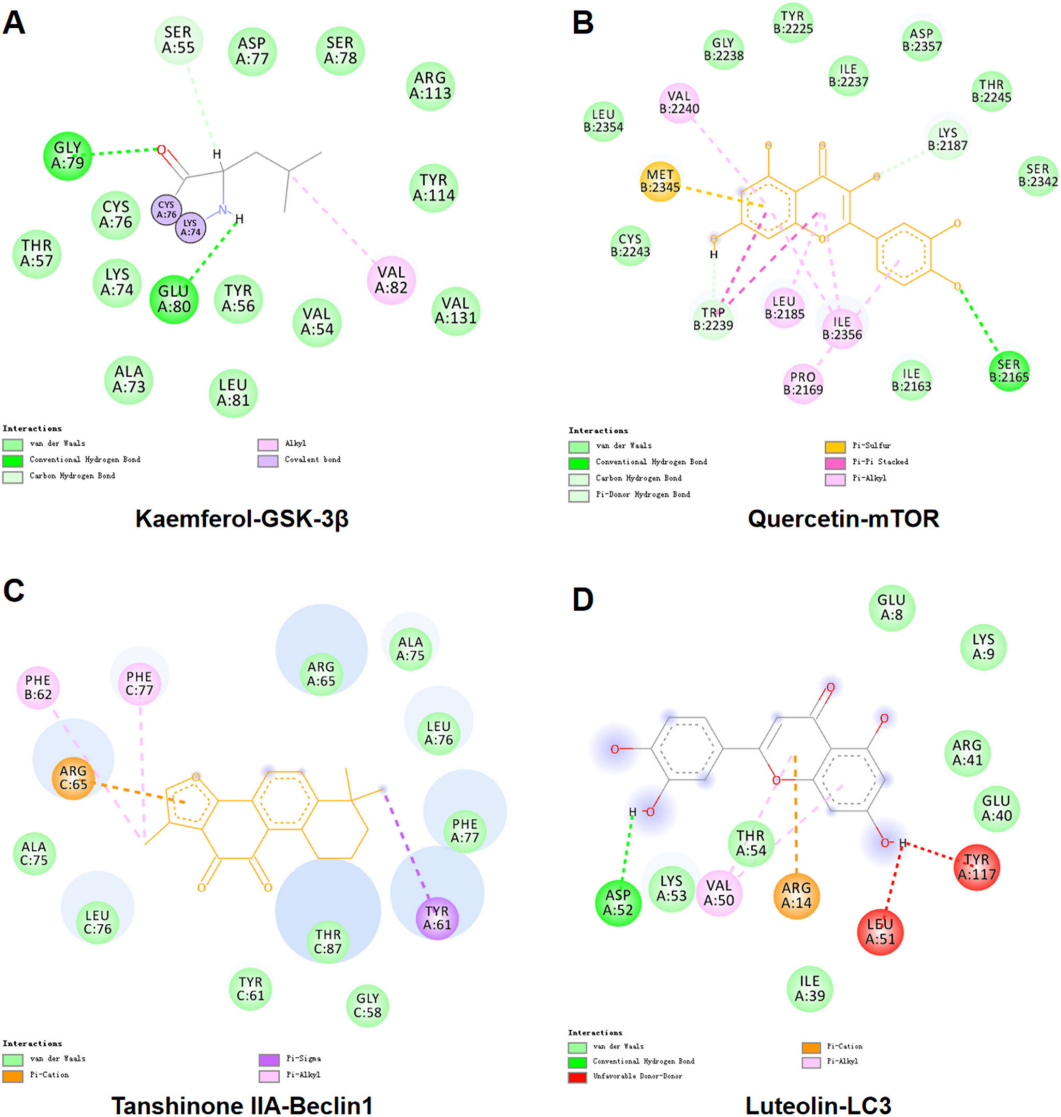
The interaction of important components with GSK-3β, mTOR, Beclin1, LC3. (A) In the active pocket of GSK-3β, *Kaempferol* formed hydrogen bonds with GLY A:79 and GLU A:80, and there was an alkyl interaction with VAL A:82. (B) *Quercetin* interacts with MET B:2345, TRP B:2239, and LEU B:2185 in mTOR to form π-sulfur, π-π stacking, and π-alkyl, respectively. (C) Docking with Beclin 1, *Tanshinone* IIA forms π-cation, π-σ and π-alkyl interactions with ARG C:65, TYR A:61, and PHE B:62, respectively. (D) In addition to hydrogen bonding with ASP A:52, *Luteolin* also forms π-cation interaction with ARG A:14 and π -alkyl interaction with VAL A:50 in the binding of LC3.

KEGG analysis revealed that TGC might improve MIRI by regulating autophagy-related proteins in the mTOR pathway. To further verify the interaction of TGC with targets in this pathway (Figure 5), we performed the docking of important compounds with the following proteins: GSK-3β (PDB: 1H8F), mTOR (PDB: 4JSV), Beclin1 (PDB: 6HOJ), and LC3 (PDB: 7ELG). The value of affinity reflected the strength of docking. As shown in Table 1, the affinity values were all <-5 kcal/mol, suggesting that TGC may interact with autophagy-related proteins in the mTOR pathway to improve MIRI.

**Table 1.** Docking score of important compounds with GSK-3β, mTOR, Beclin1, and LC3.

Molecular	GSK-3β (kcal/mol)	mTOR (kcal/mol)	Beclin1 (kcal/mol)	LC3 (kcal/mol)
Quercetin	−9	−7.8	−8.6	−6.5
Tanshinone IIA	−9.5	−9	−9.7	−6.9
Kaempferol	−9.1	−7.9	−8.6	−6.5
Luteolin	−8.8	−8.1	−8.6	−6.4
Bifendate	−7.6	−6.4	−8.2	−5.5
Przewalskin b	−9.3	−8	−9.1	−6.1
Mono-*O*-methylwightin	−8.6	−7.6	−7.8	−5.2
2-(4-Hydroxy-3-methoxyphenyl)-5-(3-hydroxypropyl)-7-methoxy-3-benzofurancarboxaldehyde	−8	−8	−8.4	−5.6

### Effect of TGC on MIRI heart

**Figure 6.**
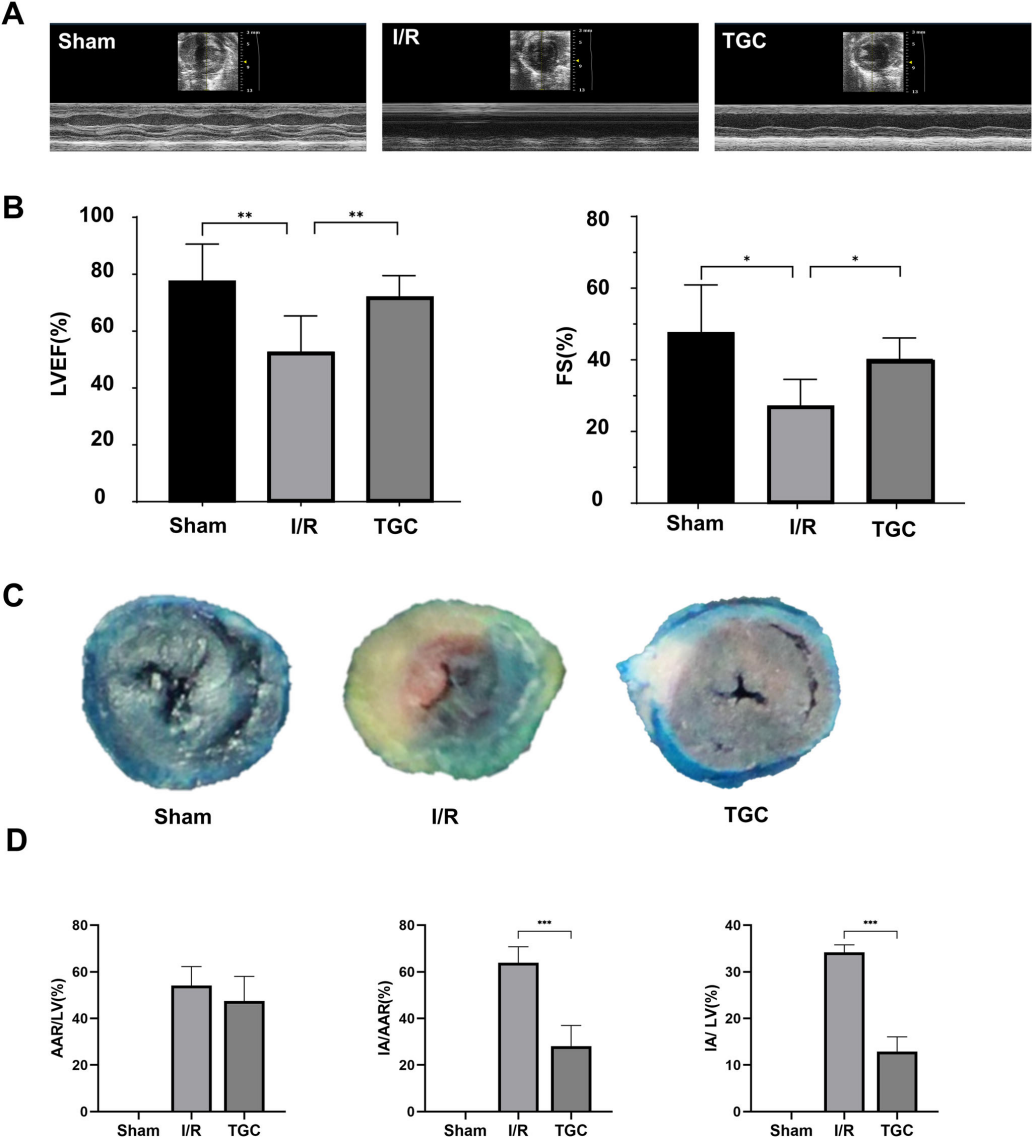
Results of each group in echocardiography and Evans blue/TTC staining. (A) Representative images of M-mode echocardiography after MIRI. (B) LVEF and FS measured according to echocardiography (*n* = 6). (C) Representative images of Evans blue/TTC staining. (D) The area of myocardial infarction was measured according to Evans blue/TTC staining (*n* = 3). *p < 0.05, **p < 0.01, ***p < 0.001.

Echocardiography showed abnormal ventricular wall motion in mice after surgery (Figure 6(A)). Compared with sham group, LVEF and FS in I/R group decreased significantly (*p* < 0.05). After TGC treatment, these two parameters were significantly increased (*p* < 0.05), suggesting that TGC was beneficial to improve cardiac function after MIRI (Figure 6(B)).

In Evans Blue/TTC staining (Figure 6C), there was no significant difference in AAR/LV between TGC group and I/R group. IA/LV and IA/ARR in the TGC group were significantly lower than those in I/R group (*p* < 0.05), indicating that TGC could reduce the proportion of myocardial infarction area (Figure 6(D)).

### TGC reduce the apoptosis of H/R cells

**Figure 7.**
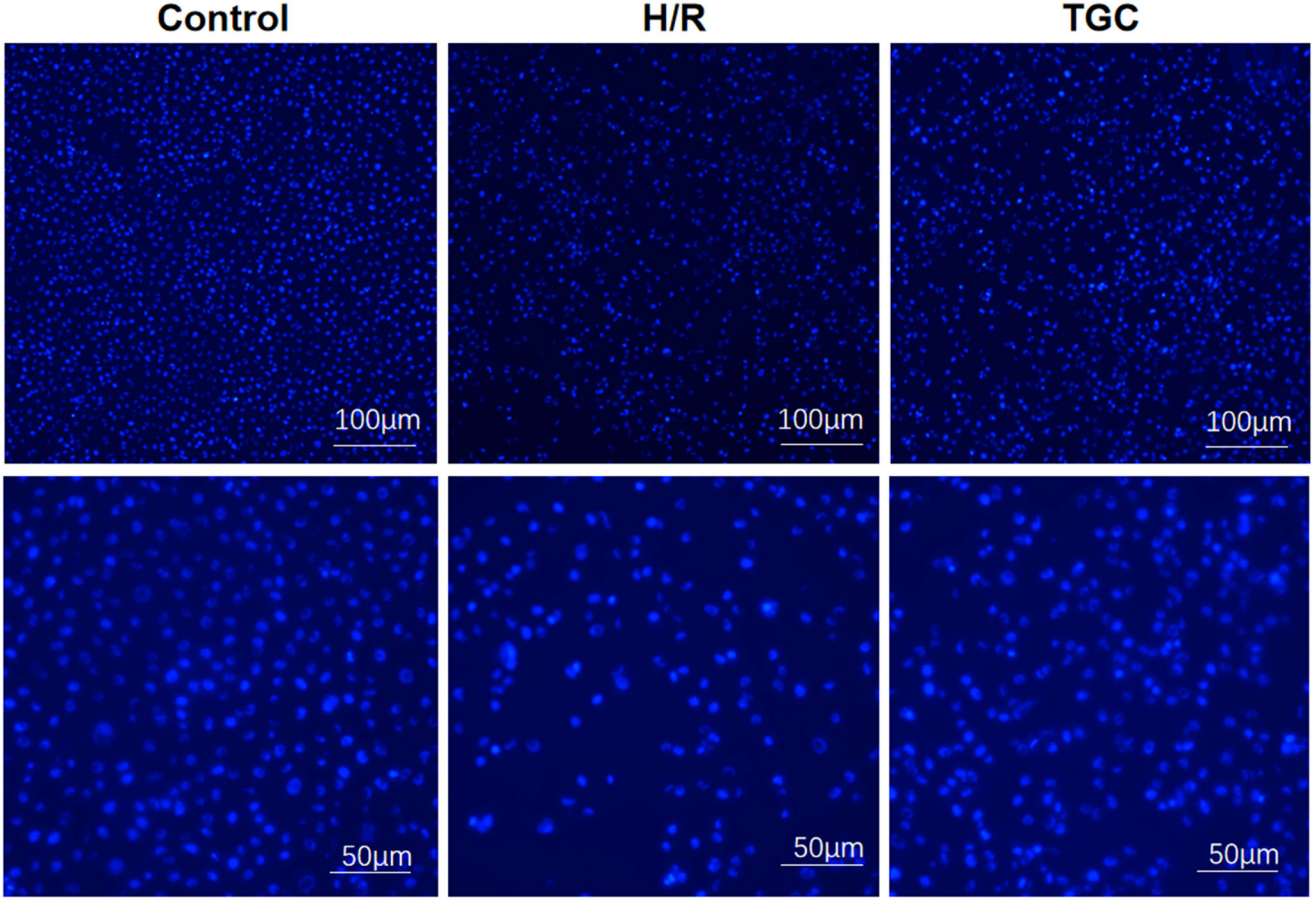
Hoechst 33258 staining in H/R cell.

In Hoechst 33258 staining, bright blue nuclei represented apoptosis. The ratio of bright blue nuclei in TGC group was significantly lower than that in H/R group, indicating that the apoptosis rate of cells decreased after TGC treatment (Figure 7). These results suggested that TGC inhibit the apoptosis of coronary endothelial cells induced by H/R.

### TGC upregulates LC3 expression in H/R cells

**Figure 8.**
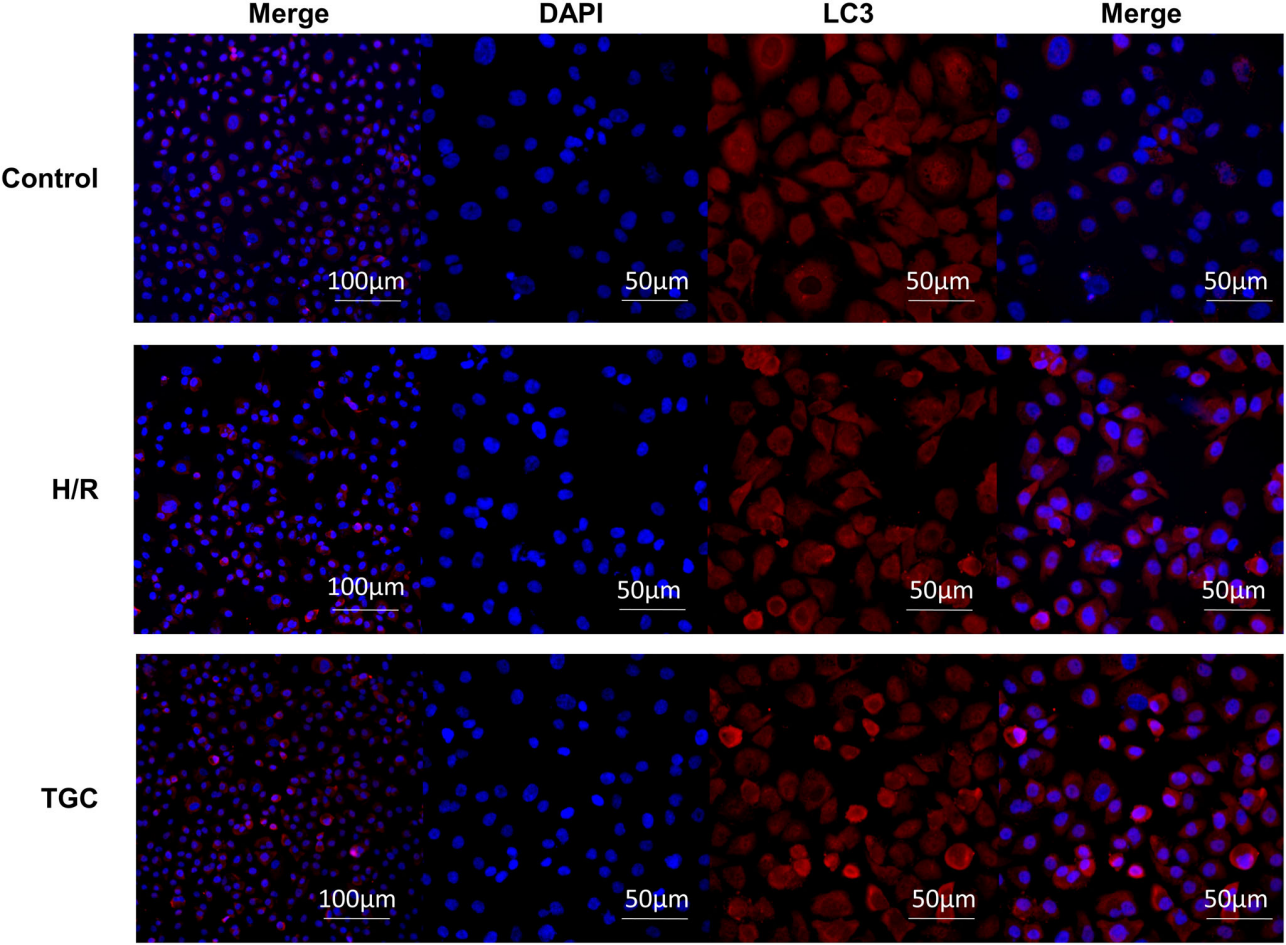
The LC3 protein expression in H/R cells. Blue fluorescence represents the nucleus, and red fluorescence represents LC3.

To clarify the role of autophagic proteins, we performed immunofluorescence detection of LC3. As shown in Figure 8, the level of LC3 (red fluorescence) in TGC group was significantly higher than that in H/R group, manifesting that TGC may inhibit apoptosis by enhancing autophagy.

### TGC inhibits apoptosis by regulating autophagy-related proteins in the mTOR pathway

**Figure 9.**
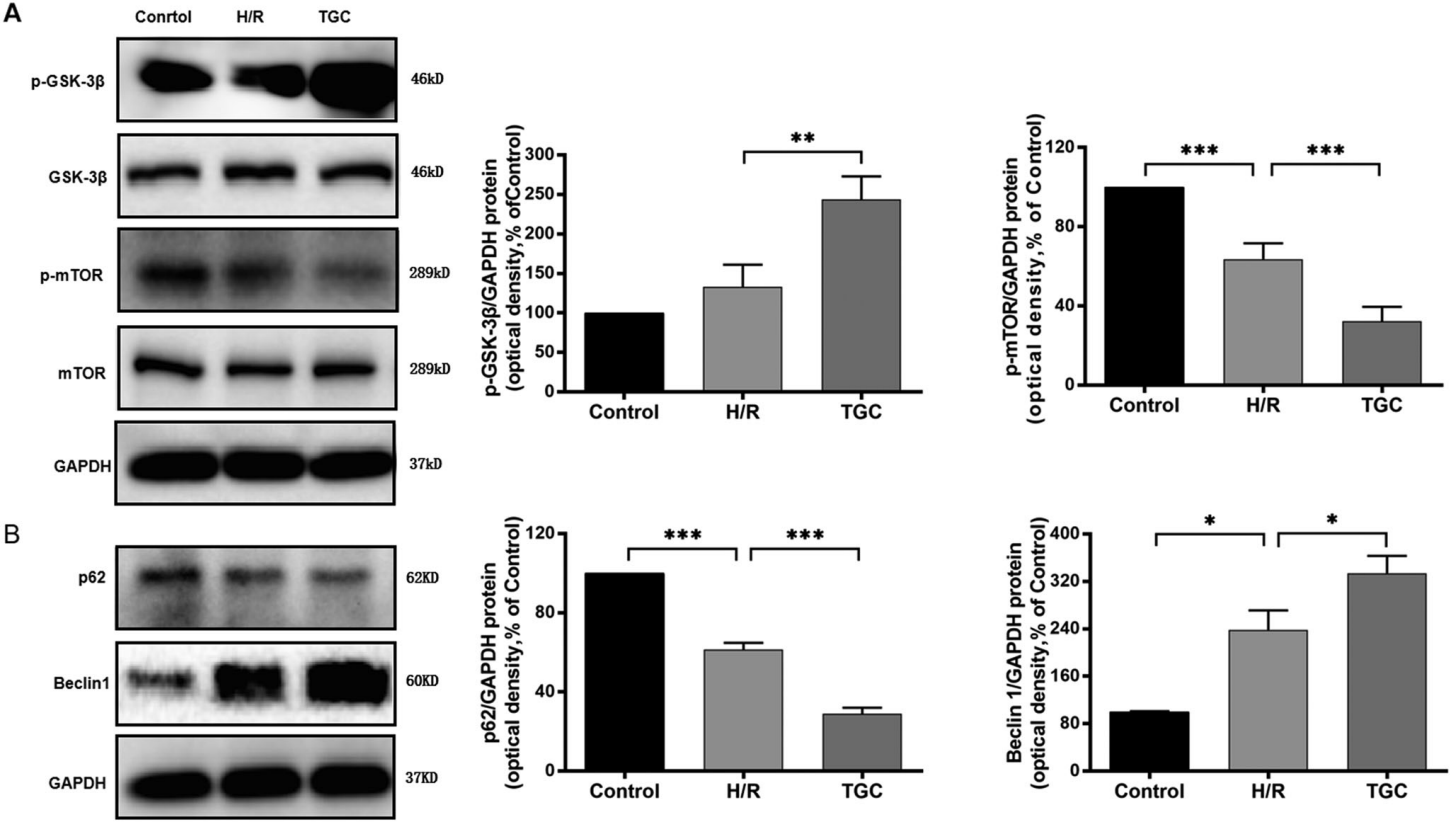
The expression of autophagy-related proteins in the mTOR pathway. (A) TGC decreased the level of p-mTOR and increased p-GSK-3β, which was assessed by Western blot. (B) TGC reduced the level of p62 and increased Beclin1, which was assessed by Western blot. *N* = 3. *p < 0.05, **p < 0.01, ***p < 0.001.

As shown in western blot (Figure 9), after treatment with TGC, the levels of p-GSK-3 β and Beclin1 increased, while p-mTOR and p62 were decreased compared with those in H/R group (*p* < 0.05). It was further suggested that TGC inhibited apoptosis of coronary endothelial cells by regulating autophagy-related proteins in the mTOR pathway.

## Discussion

Network pharmacology analysis suggested that the important active ingredients in TGC, including quercetin, luteolin, tanshinone IIA, kaempferol and bifendate improved MIRI through mTOR pathway and autophagy. Experiment has further determined that TGC reduced the area of myocardial infarction and increased the left ventricular ejection fraction, by regulating autophagy-related proteins in the mTOR pathway to inhibit cell apoptosis.

Previous studies have shown that the level of autophagy affected the repair of ischemia-reperfusion injury (Zhang et al. 2019c). Autophagy enhances the survival of cardiomyocytes after I/R injury by removing damaged mitochondria and other cellular contents (Khuanjing et al. 2021). In the case of hypoxia or lack of nutrient substrates, autophagy provides energy by degrading impaired organelles, thereby exerting a cytoprotective effect (Varshavsky 2017; Yu et al. 2018; Yang et al. 2019). Numerous cardiovascular diseases are associated with endothelial dysfunction, and regulating autophagy may be a novel frontier in the treatment of these conditions (Schaaf et al. 2019; Hughes et al. 2020; Shan et al. 2021; Mameli et al. 2022).

Autophagy initiation is regulated by multiple signaling pathways (Dikic and Elazar 2018; Levine and Kroemer 2019). mTOR is a relatively conserved serine/threonine-protein kinase that regulates various biological functions, including cell division, differentiation, apoptosis, and autophagy (Wang and Proud 2006; Saxton and Sabatini 2017; El Hiani et al. 2019). Under oxidative stress, mTOR promotes tissue repair by regulating the process of autophagy-related proteins, such as beclin1, p62, and LC3 (Tang et al. 2017; Zhou et al. 2018; Al-Bari and Xu 2020). During I/R, the mTOR signaling pathway is inhibited, while autophagy is activated, thus increasing the level of Beclin1 (Dai et al. 2017). In addition, Beclin1 knockout prevents the production of autophagosomes, promoting apoptosis and tissue damage (Dai et al. 2017).

As a conserved serine/threonine kinase, GSK-3β exerts biological effects by controlling mTOR (Urbanska et al. 2018). By regulating the expression of GSK-3β and mTOR, autophagy signals are induced to decrease myocardial infarct size and promote cardiac recovery in MIRI mouse models (Li et al. 2014).

The important active components of TGC have a role in protecting the heart by regulating autophagy. Quercetin alleviates oxidative stress-induced damage and restores mitochondrial functions by enhancing autophagy (Daw and Law 2021). Moreover, luteolin improves cardiac function, by upregulating autophagy and inhibiting inflammation (Hu et al. 2016; Wu et al. 2020). Additionally, tanshinone IIA induces autophagy by regulating the mTOR signaling pathway to reduce myocardial cell apoptosis (Zhang et al. 2019b). Kaempferol upregulates GSK-3β to inhibit the mTOR pathway, increasing LC3-II and Beclin 1, for alleviating ox-LDL-induced apoptosis (Che et al. 2017; Hoang et al. 2019).

Molecular docking results indicated that the important compound had a high affinity for the target, suggesting that TGC may protect the heart through autophagy proteins associated with the mTOR pathway. Based on autophagy, exploring the mechanism of TGC in the treatment of MIRI could further clarify the drug target, which was conducive to the research and development of novel medicine for coronary artery disease.

## Conclusions

**Figure 10.**
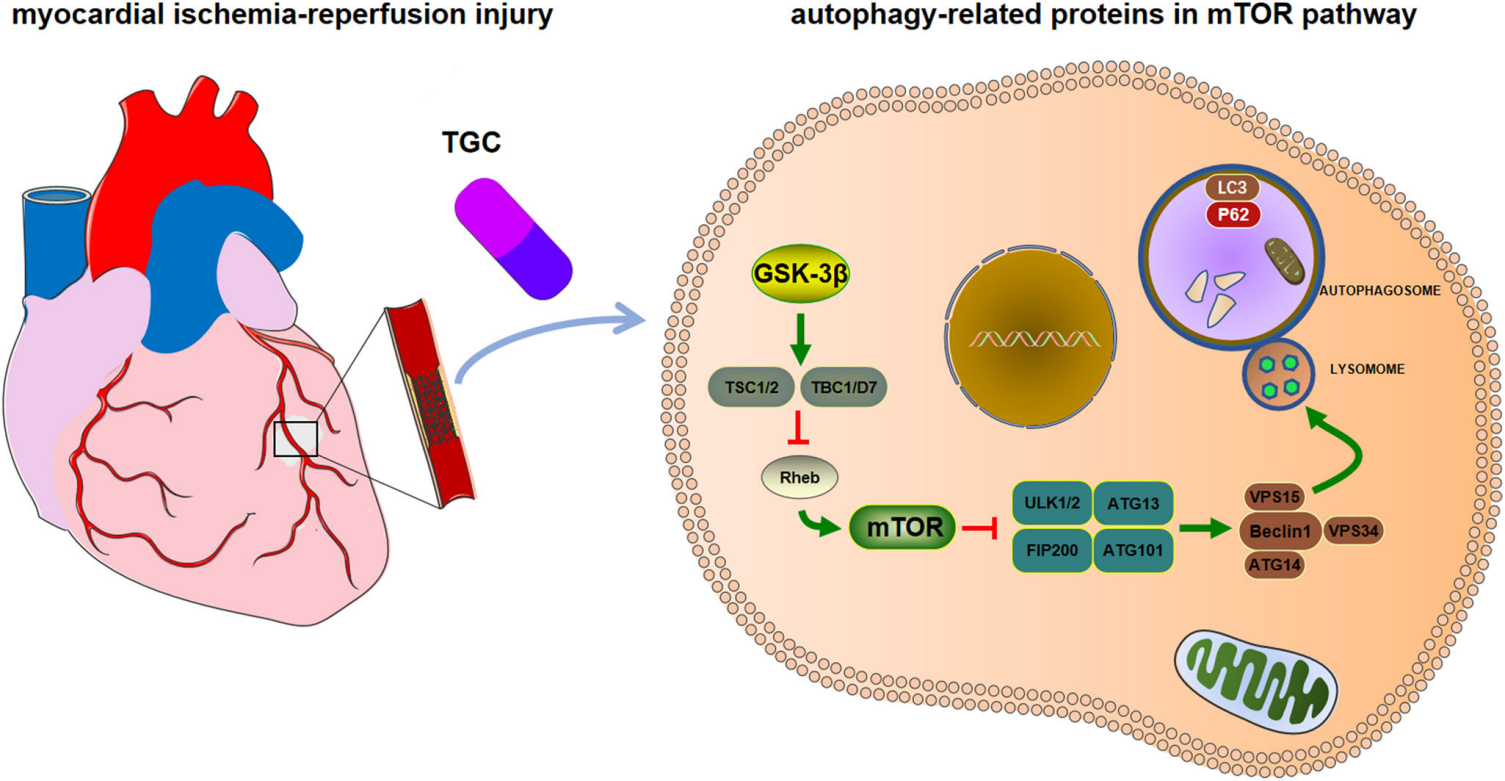
The interaction of autophagy-related proteins in the mTOR signaling pathway in MIRI. Green lines represent activation, while red lines refer to inhibition. (Reference: KEGG database).

Based on network pharmacology and experimental studies, we found that TGC exerted a cardioprotective effect against MIRI, by upregulating autophagy-related proteins through mTOR pathway (Figure 10). These data suggested that TGC may be a therapeutic option for MIRI. However, the present study had certain limitations. Insufficient databases were utilized in network pharmacology analysis, resulting in omissions in data collection. In addition, randomized controlled trials are needed for further validation for clinical application.

## Data Availability

The raw data of this study were available upon request.
